# Prognostic Implication of Physical Signs of Congestion in Acute Heart Failure Patients and Its Association with Steady-State Biomarker Levels

**DOI:** 10.1371/journal.pone.0096325

**Published:** 2014-05-06

**Authors:** Sayoko Negi, Mitsuaki Sawano, Shun Kohsaka, Taku Inohara, Yasuyuki Shiraishi, Takashi Kohno, Yuichiro Maekawa, Motoaki Sano, Tsutomu Yoshikawa, Keiichi Fukuda

**Affiliations:** 1 Department of Cardiology, Keio University School of Medicine, Tokyo, Japan; 2 Sakakibara Heart Institute, Tokyo, Japan; Emory University, United States of America

## Abstract

**Background:**

Congestive physical findings such as pulmonary rales and third heart sound (S3) are hallmarks of acute heart failure (AHF). However, their role in outcome prediction remains unclear. We sought to investigate the association between congestive physical findings upon admission, steady-state biomarkers at the time of discharge, and long-term outcomes in AHF patients.

**Methods:**

We analyzed the data of 133 consecutive AHF patients with an established diagnosis of ischemic or non-ischemic (dilated or hypertrophic) cardiomyopathy, admitted to a single-center university hospital between 2006 and 2010. The treating physician prospectively recorded major symptoms and congestive physical findings of AHF: paroxysmal nocturnal dyspnea, orthopnea, pulmonary rales, jugular venous distension (JVD), S3, and edema. The primary endpoint was defined as rehospitalization for HF.

**Results:**

Majority (63.9%) of the patients had non-ischemic etiology and, at the time of admission, S3 was seen in 69.9% of the patients, JVD in 54.1%, and pulmonary rales in 43.6%. The mean follow-up period was 726 ± 31days. Patients with pulmonary rales (p < 0.001) and S3 (p  =  0.011) had worse readmission rates than those without these findings; the presence of these findings was also associated with elevated troponin T (TnT) levels at the time of discharge (odds ratio [OR] 2.8; p  =  0.02 and OR 2.6; p  =  0.05, respectively).

**Conclusion:**

Pulmonary rales and S3 were associated with inferior readmission rates and elevated TnT levels on discharge. The worsening of the readmission rate owing to congestive physical findings may be a consequence of on-going myocardial injury.

## Introduction

The evaluation of acute heart failure (AHF) patients starts with careful history taking and physical examination. Signs of congestion and findings related to pulmonary rales, third heart sound (S3), and jugular venous distention (JVD) are known to have important diagnostic importance for AHF patients. However, the association between congestive physical findings in AHF patients and their clinical outcomes has not been well established [1]. In addition, the exact reason why these congestive physical findings are related to adverse clinical outcomes is still unclear.

In the modern management of AHF, biomarkers are used because they are thought to reflect common pathological abnormalities such as acute myocardial injury [2]–[6] or volume overload in the left ventricle [7]–[9]. The levels of these biomarkers are measured to obtain “subclinical” pathological and additional prognostic information. Therefore, we sought to examine the association between congestive physical findings upon admission, steady-state biomarker levels at the time of discharge, and long-term outcomes in AHF patients. Clarification of the role of congestive physical findings will aid in risk stratification of AHF patients in a cost-effective manner.

## Methods

### Study Subjects

This study registered AHF patients admitted to a single-center tertiary hospital between 2006 and 2010, for treatment of AHF, which was diagnosed according to the Framingham criteria. From a total of 339 patients, 110 patients (32.4%) were excluded from the final analysis because of incomplete data about physical findings on admission, biomarker levels on discharge, dates of admission and discharge, outcomes, or underlying conditions; and 96 patients (28.3%) were excluded because of diagnoses other than ischemic cardiomyopathy (ICM) or non-ischemic cardiomyopathy such as dilated cardiomyopathy (DCM) or hypertrophic cardiomyopathy (HCM). The final study population consisted of 133 patients. Informed consent was obtained from each patient, and written consent was also obtained from all of the participants in the study.

DCM was defined as echocardiographic demonstration of unexplained left ventricular (LV) dilatation (i.e., LV diastolic dimension ≥55 mm) and impaired contraction (i.e., LV ejection fraction <45%) without the presence of obstruction coronary disease. ICM was defined as LV ejection fraction <40% with previously known myocardial infarction or evidence of severe coronary disease on coronary angiography. HCM was defined as presence of increased LV wall thickening ≥15 mm in the absence of identifiable cause for LVH such as hypertension or valvular heart disease. All patients with non-ischemic cardiomyopathy underwent coronary angiography and cardiac biopsy to rule out obstructive coronary disease and infiltrative heart disease. The study protocol conformed to the ethical guidelines of the 1975 Declaration of Helsinki, and the study was approved by Institutional Review Board of Keio University School of Medicine.

### Verification of Physical Findings and Measurement of Cardiac Biomarkers

Internal medicine residents obtained all physical findings of patients upon admission and coded the findings in pre-specified case report form. The physical findings were consisted with paroxysmal nocturnal dyspnea, orthopnea, pulmonary rales, JVD, and S3. Cardiology physicians verified these findings subsequently when the patients were transferred to in-patient service.

Plasma brain natriuretic peptide (BNP) levels were measured at the time of admission as well as discharge, using a commercially available assay kit (Shionogi, Tokyo, Japan). Cardiac troponin T (cTnT) level was measured at the time of discharge (Roche Diagnostics, Tokyo, Japan). The lower limit for detection of cTnT was 0.01 ng/mL. Serum creatinine levels were determined by standard laboratory methods. Clinical data were obtained by interviewing patients and from hospital medical records.

### Follow-up and Endpoints

The study endpoint was rehospitalization for AHF. The treating physicians made decisions under the usual standard of care. In most cases, patients were readmitted when clinical signs of decompensation such as orthopnea or lower extremity edema were present. The mean length of stay for the index hospitalization was 19 ± 13 days. The mean delay for the initial outpatient follow-up visit upon discharge was 14 ± 10 days (16 patients were transferred to a different hospital, one patient died during hospitalization, one patient was transferred to a different department, and 13 were unknown). The data regarding endpoints were available for all the patients and the mean follow-up period was 726 ± 31 days. Follow-up data were obtained from either the medical records or direct inquiry with the patients or patients' family by mail or phone.

### Statistical Analysis

The study population was divided into two groups: readmission group and non-readmission group. Low ejection fraction (EF) was defined as EF <40% and elevated TnT level was defined as a serum TnT concentration of >0.1 ng/mL. Chi-square tests were used to compare categorical variables and student *t*-tests were used for continuous variables. Categorical variables were expressed as numbers (percentages) and continuous variables were expressed as the mean standard deviation.

Kaplan-Meier event curves were constructed and compared using the log-rank test. Multivariable Cox proportional hazards models were constructed using categorical variables that were statistically significant according to univariate analysis. Outcomes were adjusted for age, sex, BNP, and systolic blood pressure on admission. Finally, logistic regression analysis was used to quantify the association between each finding and biomarker level. Statistical analysis was performed using the SPSS software, version 19.0 (SPSS Inc., Chicago, Illinois). Significance was set at a p value of 0.05.

## Results

The 133 patients enrolled in this study had a mean age of 65.4 ± 15.2 years, and 22.6% were women. Majority (63.9%) of the patients had non-ischemic etiology: 57.1% had DCM and 6.7% had HCM. At the time of admission, the mean systolic blood pressure (sBP) was 131.8 ± 30.8 mmHg. As for the congestive physical findings, 69.9% had S3, 56.4% had edema, 54.1% had JVD, and 43.6% had pulmonary rales. Details of the characteristics of the study subjects against the presence and absence of each finding are shown in Table 1. Among the patients who were readmitted for AHF, rales were seen in none (0%, no documentation in 21 patients [15.8%]), S3 were ten (21.3%, no documentation in 13 patients [9.7%]), edema were two (4.3%, no documentation in 36 patients [27.1%]).

**Table 1:** Characteristics of the study subjects.

		Readmission for ADHF (+) (n = 47)	Readmission for ADHF (−) (n = 86)	P value
Age, yrs		70.21 ± 1.76	62.78 ± 1.73	0.001
Women, %		21.3 (n = 10)	23.3 (n = 20)	0.366
Comorbidities	Ischemic heart disease, %	36.2 (n = 17)	36.0 (n = 31)	0.931
	Dilated cardiomyopathy, %	57.4 (n = 27)	57.0 (n = 49)	0.958
	Hypertrophic cardiomyopathy, %	6.4 (n = 3)	7.0 (n = 6)	0.892
AF, %		36.2 (n = 17)	27.1 (n = 23)	0.655
Systolic Blood Pressure, mmHg		132.80 ± 4.98	131.27 ± 3.16	0.530
NYHA functional class	on admission	3.07 ± 0.11	3.02 ± 0.08	0.705
	on discharge	2.07 ± 0.04	1.98 ± 0.02	0.031
BNP, pg/mL	on admission	770.36 ± 98.26	682.62 ± 79.61	0.030
	on discharge	395.51 ± 49.90	331.59 ± 60.96	0.055
BUN, mg/dL	on admission	24.59 ± 10.42	20.75 ± 9.30	0.041
	on discharge	28.80 ± 14.09	23.35 ± 12.02	0.031
Left ventricular ejection fraction ≤40%, %		48.9 (n = 23)	41.9 (n = 36)	0.541
Troponin T level on discharge >0.10 ng/mL, %		37.2 (n = 16)	22.1 (n = 17)	0.004
Death, %		18.2 (n = 8)	4.7 (n = 4)	0.001
Physical signs on admission	PND, %	40.4 (n = 19)	30.2 (n = 26)	0.488
	Orthopnea, %	34.0 (n = 16)	35.3 (n = 30)	0.624
	JVD, %	61.7 (n = 29)	50.6 (n = 43)	0.088
	Edema, %	53.2 (n = 25)	58.1 (n = 50)	0.982
	Rales, %	56.5 (n = 26)	37.6 (n = 32)	0.003
	S3, %	80.9 (n = 38)	64.0 (n = 55)	0.054
Physical signs on discharge	Edema, %	4,3 (n = 2)	4.7 (n = 4)	0.989
	Rales, %	0.0 (n = 0)	2.3 (n = 2)	0.316
	S3, %	21.3 (n = 10)	25.6 (n = 22)	0.800
Medicine on admission	Spironolactone, %	34.0 (n = 16)	36.0 (n = 31)	0.862
	ACE inhibitor, %	29.8 (n = 14)	23.3 (n = 20)	0.245
	ARB inhibitor, %	25.5 (n = 12)	32.6 (n = 28)	0.620
	β-blocker, %	57.4 (n = 27)	50.0 (n = 43)	0.156

Patients readmitted for ADHF were significantly younger and had higher NYHA class on discharge, BNP levels on admission, BUN levels on admission and discharge, TnT levels on discharge, and death rates than those not readmitted for ADHF (p  =  0.001, 0.031, 0.030, 0.041, 0.031, 0.004 and 0.001, respectively). The proportion of patients with rales was significantly higher in the readmission group than in the non-readmission group (p  =  0.003). Missing values were considered to be negative about physical signs on discharge (missing values of patients with rales were 21, S3 were 13, edema were 36).

ADHF  =  acute decompensated heart failure, AF  =  atrial fibrillation, NYHA  =  New York Heart Association, BNP  =  brain natriuretic peptide, BUN  =  blood urea nitrogen, PND  =  paroxysmal nocturnal dyspnea, JVD  =  jugular venous distention, S3  =  the third heart sound, ACE  =  angiotensin-converting enzyme, ARB  =  angiotensin II receptor blockers

We performed an additional analysis to see whether patients receiving guideline-based HF medication (e.g., spironolactone, angiotensin-converting enzyme inhibitor, angiotensin II receptor blockers, or β-blocker) prior to admission would have difference in the prevalence of HF-related physical findings compared to those not receiving guideline-based treatment. As shown in Table S1A in Tables S1, there was no significant difference in the readmission rate between patients on each of the optimal guideline based medications before admission and after admission. Individually, patients on beta-blockers had lower percentage of JVD, edema and S3 (p = 0.042, 0.010 and 0.028, respectively). Patients on spironolactone before admission had lower percentage of PND, edema and rales (p = 0.023, 0.004 and 0.002, respectively). We believe that these findings suggest that there might be a possible effect of optimal medical therapy altering physical findings on admission. At the same time, however, we would like to note that these differences did not show significant effect on primary outcome (Table 1B).

**Figure 1:**
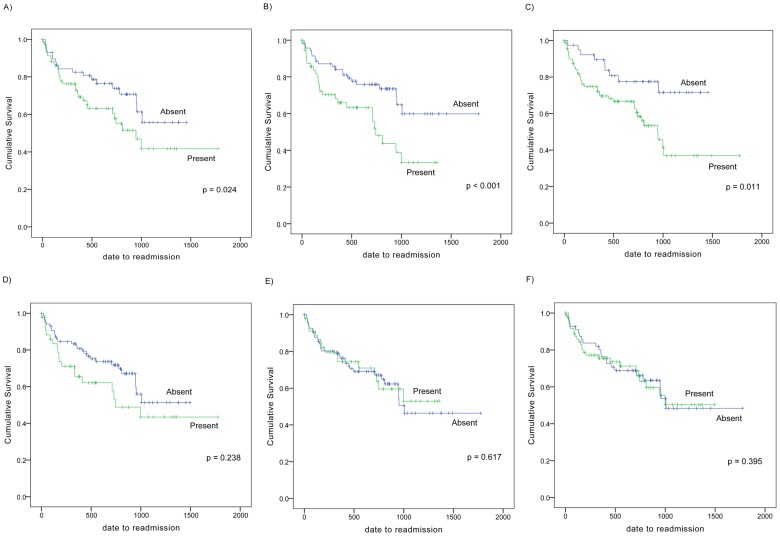
Kaplan Meier analysis of event-free survival according to the presence or absence of physical findings. Jugular venous distension (JVD) (A), rales (B), third heart sound (S3) (C), paroxysmal nocturnal dyspnea (D), orthopnea (E), and edema (F). Patients with JVD, pulmonary rales, or S3 had worse readmission rates that those without these findings (log-rank p  =  0.024, p < 0.001, p  =  0.001, respectively).

Combined endpoint of rehospitalization and death was met in 37.0% of the patients, and the overall mortality rate was 11.6%. With regard to the congestive physical findings, patients who presented with JVD, pulmonary rales, and S3 had worse readmission rates than those without these findings (Figure 1; log-rank p  =  0.024, p < 0.001 and p  =  0.011, respectively). Predictors of readmission rate are listed in Table 2. Notably, the presence of pulmonary rales (hazard ratio [HR] 2.03, 95% confidence interval [CI] 1.09–3.79; p  =  0.026) and S3 (HR 2.05, 95%CI 1.12–3.75; p  =  0.019) at the time of admission were related to readmission even after adjustment for age, sex, BNP, and sBP.

**Table 2:** Predictors associated with readmission rate.

Predictors		HR (95% CI)	P value	HR Adjusted for Age and Sex (95% CI)	P value	HR Adjusted for Age, Sex, BNP level, and sBP on admission (95% CI)	P value
Age		1.026 (1.010–1.043)	0.002	–	–	–	–
Women, %		1.348 (0.795–2.287)	0.268	–	–	–	–
Comorbidities	Ischemic heart disease, %	0.982 (0.604–1.596)	0.982	–	–	–	–
	Dilated cardiomyopathy, %	1.002 (0.792–1.268)	0.986	–	–	–	–
	Hypertrophic heart disease, %	1.021 (0.729–1.430)	0.905	–	–	–	–
AF, %		1.191 (0.714–1.987)	0.502	–	–	–	–
Systolic Blood Pressure, mmHg		0.997 (0.989–1.006)	0.503	–	–	–	–
Systolic Blood Pressure ≥140, mmHg		0.811 (0.483–1.364)	0.430	–	–	–	–
NYHA functional class	on admission	1.113 (0.816–1.518)	0.499	–	–	–	–
	on discharge	3.064 (1.141–8.232)	0.026	–	–	–	–
BNP, pg/mL	on admission	1.001 (1.000–1.001)	<0.001	–	–	–	–
	on admission ≥150	8.936 (2.185–36.555)	0.002	–	–	–	–
	on discharge	1.001 (1.001–1.001)	<0.001	–	–	–	–
	on discharge ≥150	2.610 (1.440–4.731)	0.002	–	–	–	–
Left ventricular ejection fraction ≤40%, %		1.166 (0.729–1.866)	0.522	–	–	–	–
Troponin T on discharge >0.10 ng/mL, %		2.404 (1.449–3.986)	0.001	–	–	–	–
PND		1.351 (0.819–2.228)	0.239	1.174 (0.706–1.952)	0.535	1.167 (0.668–2.037)	0.587
Orthopnea		0.867 (0.496–1.517)	0.618	0.727 (0.408–1.294)	0.278	0.690 (0.378–1.260)	0.227
JVD		1.819 (1.074–3.078)	0.026	1.513 (0.869–2.632)	0.143	1.396 (0.783–2.488)	0.258
Edema		1.246 (0.750–2.069)	0.396	1.077 (0.634–1.830)	0.783	1.055 (0.597–1.863)	0.855
Rales		2.489 (1.479–4.191)	0.001	1.951 (1.096–3.474)	0.023	2.034 (1.090–3.794)	0.026
S3		2.077 (1.165–3.700)	0.013	1.908 (1.064–3.419)	0.030	2.056 (1.126–3.754)	0.019

Predictors associated with readmission rate according to multivariable Cox proportional hazards models. Even after adjusting for age, sex, BNP level, and sBP on admission, the presence of rales and S3 were significantly related to the readmission rate (p  =  0.026 and 0.019, respectively).

HR  =  hazard ratio, sBP  =  systolic blood pressure, AF  =  atrial fibrillation, NYHA  =  New York Heart Association, BNP  =  brain natriuretic peptide,

PND  =  paroxysmal nocturnal dyspnea, JVD  =  jugular venous distention, S3  =  the third heart sound

Table 3 shows the association between biomarker levels at the time of discharge and AHF physical findings. The presence of pulmonary rales was associated with high BNP levels (≥150 pg/mL) at the time of discharge (OR 2.208, 95%CI 1.077–4.525; p  =  0.031), although this association was found to be insignificant after adjustment for age and sex. Presence of edema (OR 3.758, 95%CI 1.719–8.212; p  =  0.001), rales (OR 3.990, 95%CI 1.869–8.516; p < 0.001), and S3 (OR 2.939, 1.206–7.159; p  =  0.018) were related to high TnT levels (>0.10 ng/mL). Pulmonary rales (OR 2.874, p  =  0.017) and S3 (OR 2.614, p  =  0.050) were significantly associated with high TnT levels after adjustment for age, sex, sBP, and BNP on admission.

**Table 3:** Logistic regression analysis.

			A. Predictors of BNP level on discharge ≥150 pg/mL			
Variables	OR (95% CI)	P value	OR Adjusted for Age and Sex (95% CI)	P value	OR Adjusted for Age, Sex, BNP level, and sBP on admission (95% CI)	P value
PND	0.901 (0.448–1.811)	0.770	0.732 (0.352–1.521)	0.403	0.545 (0.237–1.252)	0.153
Orthopnea	1.023 (0.492–2.125)	0.952	0.963(0.456–2.036)	0.922	0.757 (0.330–1.735)	0.511
JVD	1.488 (0.767–2.889)	0.240	1.237 (0.617–2.479)	0.548	0.559 (0.242–1.288)	0.172
Edema	1.397 (0.717–2.725)	0.326	1.235 (0.622–2.454)	0.546	0.841 (0.379–1.870)	0.671
Rales	2.208 (1.077–4.525)	0.031	1.689 (0.790–3.609)	0.176	1.473 (0.625–3.473)	0.376
S3	1.852 (0.940–3.650)	0.075	1.677 (0.836–3.364)	0.146	1.656 (0.741–3.702)	0.219
			B. Predictors of troponin T on discharge >0.10 ng/mL			
Variables	OR (95% CI)	P value	OR Adjusted for Age and Sex (95% CI)	P value	OR Adjusted for Age, Sex, BNP level, and sBP on admission (95% CI)	P value
PND	1.368 (0.663–2.822)	0.397	1.119 (0.521–2.406)	0.773	1.054 (0.475–2.337)	0.897
Orthopnea	0.870 (0.394–1.919)	0.730	0.750 (0.325–1.729)	0.499	0.654 (0.273–1.567)	0.340
JVD	1.773 (0.854–3.678)	0.124	1.155 (0.522–2.553)	0.722	1.043 (0.449–2.424)	0.922
Edema	3.758 (1.719–8.212)	0.001	3.019 (1.333–6.838)	0.008	3.134 (1.302–7.546)	0.011
Rales	3.990 (1.869–8.516)	<0.001	2.548 (1.127–5.760)	0.025	2.583 (1.097–6.082)	0.030
S3	2.939 (1.206–7.159)	0.018	2.547 (1.000–6.490)	0.050	2.459 (0.941–6.421)	0.066

Logistic regression analysis quantifying the association between biomarkers on discharge and physical findings and symptoms. Although the level of BNP on discharge (A) were not related to any physical findings and symptoms, the findings of edema, rales, and S3 were related to TnT levels on discharge (B) after adjusting for age, sex, BNP level, sBP on admission.

BNP  =  brain natriuretic peptide, OR  =  odds ratio, sBP  =  systolic blood pressure, PND  =  paroxysmal nocturnal dyspnea, JVD  =  jugular venous distention,

S3  =  the third heart sound

## Discussion

In our single-institution based HF registry, the presence of pulmonary rales and S3 on admission was associated with HF readmission rate and high TnT levels on discharge. These physical findings are harbingers for difficulty achieving adequate decongestion during AHF treatment, suggesting the involvement of underlying complex mechanisms such as on-going inflammation during the acute phase of AHF.

The prognostic implications of signs and symptoms for HF patients have been previously reported. Devroey et al reported that the presence of pulmonary rales was significantly associated with HF (p < 0.001) [10]. Drazner et al reported that the presence of S3 was associated with increased risk of rehospitalization in the Studies of Left Ventricular Dysfunction (SOLVD) trial [1]. These results implying that the left-sided signs of HF are associated with rehospitalization, are consistent with the results of our current study. On the other hand, right-sided HF physical findings such as JVD or edema did not show any statistical significant association with adverse outcomes, and this finding is in contrast with those of previous studies such as the SOLVD or ESCAPE trial [11], probably because of the difference in patient characteristics between the studies; the SOLVD and the ESCAPE trials examined chronic HF patients in the outpatient setting, whereas this study examined AHF patients at the time of admission. Patients with chronic HF who present with JVD have either residual secondary pulmonary hypertension or isolated pulmonary hypertension, both being signs of inadequate compensation. These signs may be of low significance when predicting adverse outcomes in AHF patients.

In our study, the left-sided physical signs of AHF were also related to elevated biomarker levels at the time of discharge. TnT is a sensitive and specific marker for myocyte injury [12] and has been studied in acute and chronic HF patients previously. Missov et al [13] found increased levels of circulating cTnT in patients with HF but no clinically significant signs of ischemia. This phenomenon is thought to be caused by coronary microvascular dysfunction [14]. The findings of previous studies highlight that increased TnT levels are independent markers of mortality in HF patients [4]. In the ADHERE trial, AHF patients who tested positive for elevated troponin levels had lower systolic blood pressure on admission, lower ejection fraction, and higher in-hospital mortality rates than those who tested negative [15]. BNP is another well-known and frequently used biomarker for HF diagnosis and treatment. It is a neurohormone specifically secreted from the cardiac chambers in response to hemodynamic stress. The BNP level is elevated in situations where the ventricles are dilated, hypertrophic, or subject to increased wall tension [16], [17]. The clinical validity of brain natriuretic peptide (BNP) measurements has been previously reported. Nishii M et al. reported that a high BNP level was a predictor of long-term risk in patients with non-ischemic dilated cardiomyopathy who were asymptomatic for more than six months after admission for AHF [18]. Januzzi and Troughton reported that BNP-guided therapy resulted in superior medical management compared to traditional care, mostly because of frequent and sophisticated drug adjustment [19]. In addition, BNP-guided therapy might be particularly attractive in older patients who are less physically active and in those whom symptoms are less reliable [20]. In our study, there were few patients with high BNP levels without significant physical findings before discharge. As the reviewer commented, these patients may benefit from BNP measurement to predict their long-term outcome such as readmission or death. However, caution is needed since other studies have suggested that BNP-guided HF management may have little or even a negative impact on elderly patients because of increased risk of drug-drug interactions and worsening organ failure secondary to polypharmacy [21]. Obviously, treatment strategies should be based on both physical signs and biomarkers. Physical signs are safe, cost-efficient non-invasive methods to assess the state of patients with HF. At times, physical signs may serve as unclear indices because of interobserver variation. In such situations, biomarkers could prove to be strong prognostic indices. The precise relationship between physical signs and biomarkers should be studied further.

In our cohort, higher readmission rates were seen in AHF patients with increased TnT levels but not in those with elevated BNP levels. This may be related to relatively high number of DCM patients included in our study. These patients are known to have high BNP levels even after they reach compensated state. The lack of statistical differences could have resulted from this unique cohort. On the contrary, AHF patients with elevated TnT level at discharge were those that required early readmission. Elevated TnT may be a more sensitive biomarker compared to BNP in identifying vulnerable AHF patients who require close monitoring post discharge.

Our study has several limitations. First, this study was conducted in a single-center tertiary university hospital. Therefore, a multicenter study with a large study population is needed to determine the correlation between physical findings and biomarker levels. Second, despite adjusting for known risk factors, according to the results of the Cox hazards models, residual confounding may have been caused by unmeasured and measured variables. Third, there were no standardized instructions for obtaining physical findings and this may have led to some degree of misclassification depending on the physicians. The physical examination results could have been inaccurate to some extent, and no confirmatory tests such as phonocardiography for S3 were performed during this study.

The significance of physical examination cannot be underestimated. A strong relationship has been shown between left-sided HF symptoms and elevated TnT levels. Therefore, focused bedside assessment is vital and our findings add to the prognostic importance of physical findings in AHF patients.

## Supporting Information

Tables S1 **This file includes Table S1 and S2.** Table S1. Presence of physical signs by the use of guideline-based heart failure medications on admission. All the data in the table are percentages. There was no significant difference in the readmission rate between patients receiving each of the optimal guidelinebased medications before and after admission. Individually, patients on beta-blockers had a lower percentage of jugular venous distention, edema, and S3 (P = 0.042, 0.010, and 0.028, respectively). Patients receiving spironolactone before admission had a lower percentage of paroxysmal nocturnal dyspnea, edema, and rales (P = 0.023, 0.004, and 0.002, respectively). Table S2. Readmission rate according to the use of guidelinebased heart failure medication at the time of admission.
(DOC)
