# Supplementary material for: Prognostic Implication of Physical Signs of Congestion in Acute Heart Failure Patients and Its Association with Steady-State Biomarker Levels
Source: PLoS One. 2014 May 6;9(5):e96325. doi: 10.1371/journal.pone.0096325 (PMC4011709; doi:10.1371/journal.pone.0096325)
Supplement: Tables S1 — This file includes Table S1 and S2. Table S1. Presence of physical signs by the use of guideline-based heart failure medications on admission. All the data in the table are percentages. There was no significant difference in the readmission rate between patients receiving each of the optimal guidelinebased medications before and after admission. Individually, patients on beta-blockers had a lower percentage of jugular venous distention, edema, and S3 (P = 0.042, 0.010, and 0.028, respectively). Patients receiving spironolactone before admission had a lower percentage of paroxysmal nocturnal dyspnea, edema, and rales (P = 0.023, 0.004, and 0.002, respectively). Table S2. Readmission rate according to the use of guidelinebased heart failure medication at the time of admission. (DOC) [file pone.0096325.s001.doc]

**Table S1.** **Presence of physical signs by the use of guideline-based heart failure medications on admission.** All the data in the table are percentages.　There was no significant difference in the readmission rate between patients receiving each of the optimal guideline-based medications before and after admission. Individually, patients on beta-blockers had a lower percentage of jugular venous distention, edema, and S3 (P = 0.042, 0.010, and 0.028, respectively). Patients receiving spironolactone before admission had a lower percentage of paroxysmal nocturnal dyspnea, edema, and rales (P = 0.023, 0.004, and 0.002, respectively).

| Medication | Physical Signs | Used on admission (n = 47) | Not used on admission (n = 86) | P value |
| --- | --- | --- | --- | --- |
| Spironolactone | PND | 19.1 (n = 9) | 38.4 (n =33 ) | 0.023 |
|  | Orthopnea | 27.7 (n = 13) | 38.4 (n = 33) | 0.214 |
|  | JVD | 44.7 (n = 21) | 57.0 (n = 49) | 0.175 |
|  | Edema | 38.3 (n = 18) | 64.0 (n = 55) | 0.004 |
|  | Rales | 23.4 (n = 11) | 51.2 (n = 44) | 0.002 |
|  | S3 | 68.1 (n = 32) | 68.6 (n = 59) | 0.951 |
| Medication | Physical Signs | Used on admission (n = 34) | Not used on admission (n = 99) | P value |
| ACE inhibitors | PND | 35.3 (n = 12) | 30.3 (n = 30) | 0.589 |
|  | Orthopnea | 32.4 (n = 11) | 35.4 (n = 35) | 0.751 |
|  | JVD | 55.9 (n = 19) | 51.5 (n = 51) | 0.660 |
|  | Edema | 61.8 (n = 21) | 52.5 (n = 52) | 0.350 |
|  | Rales | 41.2 (n = 14) | 41.4 (n = 41) | 0.981 |
|  | S3 | 61.8 (n = 21) | 70.7 (n = 70) | 0.333 |
| Medication | Physical Signs | Used on admission (n = 40) | Not used on admission (n = 93) | P value |
| Angiotensin receptor blockers | PND | 35.0 (n = 14) | 30.1 (n = 28) | 0.578 |
|  | Orthopnea | 45.0 (n = 18) | 30.1 (n = 28) | 0.098 |
|  | JVD | 50.0 (n = 20) | 53.8 (n = 50) | 0.690 |
|  | Edema | 45.0 (n = 18) | 59.1 (n = 55) | 0.133 |
|  | Rales | 35.0 (n = 14) | 44.1 (n = 41) | 0.329 |
|  | S3 | 60.0 (n = 24) | 72.0 (n = 67) | 0.171 |
| Medication | Physical Signs | Used on admission (n = 70) | Not used on admission (n = 63) | P value |
| β-blockers | PND | 30.0 (n = 21) | 33.3 (n = 21) | 0.680 |
|  | Orthopnea | 31.4 (n = 22) | 38.1 (n = 24) | 0.420 |
|  | JVD | 44.3 (n = 31) | 61.9 (n = 39) | 0.042 |
|  | Edema | 44.3 (n = 31) | 66.7 (n = 42) | 0.010 |
|  | Rales | 34.3 (n = 24) | 49.2 (n = 31) | 0.081 |
|  | S3 | 60.0 (n = 42) | 77.8 (n = 49) | 0.028 |

ACE = angiotensin-converting enzyme, ARB = angiotensin II receptor blockers, PND = paroxysmal nocturnal dyspnea, JVD = jugular venous distention, S3 = the third heart sound

**Table S2. Readmission rate according to the use of guideline-based heart failure medication at the time of admission.**

| Type of Medications | Readmitted during the follow-up (n = 47) | Not readmitted during the follow-up (n = 86) | P value |
| --- | --- | --- | --- |
| β-blocker, % | 57.4 (n = 27) | 50.0 (n = 43) | 0.156 |
| Spironolactone, % | 34.0 (n = 16) | 36.0 (n = 31) | 0.862 |
| ACE inhibitor, % | 29.8 (n = 14) | 23.3 (n = 20) | 0.245 |
| ARB inhibitor, % | 25.5 (n = 12) | 32.6 (n = 28) | 0.620 |

ACE = angiotensin-converting enzyme, ARB = angiotensin II receptor blockers
